# Effects of a Floor Hockey Intervention on Motor Proficiency, Physical Fitness, and Adaptive Development in Youths with Mild Intellectual Disabilities

**DOI:** 10.3390/ijerph18137059

**Published:** 2021-07-01

**Authors:** Po-Jen Hsu, Hung-Ling Yeh, Chia-Liang Tsai, Chia-Hua Chu, Fu-Chen Chen, Chien-Yu Pan

**Affiliations:** 1 Graduate Institute of Physical Education, National Taiwan Sport University, Taoyuan City 333, Taiwan; a0912024720@gmail.com; 2 Department of Physical Education, National Kaohsiung Normal University, Kaohsiung City 802, Taiwan; lin608860@gmail.com (H.-L.Y.); chiahua@nknucc.nknu.edu.tw (C.-H.C.); fcchen@nknu.edu.tw (F.-C.C.); 3 Institute of Physical Education, Health and Leisure Studies, National Cheng Kung University, Tainan City 701, Taiwan; andytsai@mail.ncku.edu.tw

**Keywords:** floor hockey exercise, motor skill, physical fitness, adaptive behavior, intellectual disability

## Abstract

This study examined the effects of a 12-week floor hockey training program on the motor proficiency, physical fitness, and adaptive development of youths with mild intellectual disabilities (IDs). A total of 54 youths with IDs were divided into two groups: a floor hockey exercise group (EG; *n* = 27; age, 16.59 ± 0.56 years) and a control group (CG; *n* = 27; age, 16.65 ± 0.63 years). The participants in the EG attended sessions of a floor hockey training program 3 times per week over a 12-week period. The CG group maintained their standard activities of daily living. The participants’ scores on the Bruininks–Oseretsky Test of Motor Proficiency, Second Edition, Brockport Physical Fitness Test, and traditional Chinese version of the teacher form of the Adaptive Behavior Assessment System, Second Edition, were obtained before and after the intervention. The results of the study indicate that the 12-week floor hockey training program significantly increased the participants’ scores for most indicators of motor proficiency (*p* < 0.01), physical fitness (*p* < 0.01), and adaptive development (*p* < 0.01). The findings provide evidence that physical activity interventions focusing on floor hockey training are a viable therapeutic option for treating youths with IDs.

## 1. Introduction

Intellectual disabilities (IDs) are characterized by considerable limitations in both intellectual functioning and adaptive behaviors [1,2]. Adaptive behavior is defined as the conceptual, social, and practical adaptive skills that a person has learned and performs in their everyday lives [3]. Many relevant studies have also recognized that children and adolescents (hereafter referred to as youths) with IDs tend to have deficits in motor proficiency [4,5,6]. These deficits may make them less willing or less likely to be physically active given that motor proficiency is fundamental to participation in various physical activities and sports [7,8]. Research has indicated that youths with IDs, especially those in secondary schools, engage in significantly less physical activity (PA) than do their counterparts in the general population [9]. Youths with IDs also exhibit low levels of physical fitness [10,11] and high rates of obesity [12,13,14], and experience substantial health disparities [9,15,16]. The global prevalence of overweight and obesity in youths with IDs is 30% to 33% [17], and the risk of developing overweight and obesity is 1.54 to 1.80 times higher among youths with IDs (aged 12 to 18 years) than among their typically developing peers [18]. In terms of health-related physical fitness levels, a cross-sectional study revealed that 71% to 91% of youths with IDs (aged 12 to 18 years) scored below the reference values (for typically developing youths) in cardiopulmonary and muscular fitness [19]. Such relatively poor fitness levels may exacerbate health disturbances and increase the rates of morbidity and mortality in the relevant population [12]. As youths transition to middle and late youthhood, healthcare professionals have the opportunity to promote health-enhancing PA that can likely be sustained throughout adulthood. Stodden et al. [20] proposed a model of motor development, indicating that motor proficiency and physical fitness were factors that influence PA engagement, and the relationships strengthened over time. Therefore, identifying effective PA approaches before and during this transitional period is critical because the functional ability of an individual with a disability at a young age can determine their functional ability in adulthood [21]. Most individuals with IDs experience functional and cognitive decline [22], along with lower physical performance, less participation in social activities, and the development of a sedentary lifestyle [23]. Therefore, PA interventions designed to improve motor proficiency as well as aspects of physical fitness and adaptive functioning in youths with IDs are necessary for the achievement of optimal health outcomes; moreover, such interventions would offer the greatest benefits for this population.

The basis for promoting PA in individuals with disabilities is that regular participation can help manage or improve health, physical function, and overall well-being [24]. The World Health Organization has recommended that all youths, with and without disabilities, should participate in moderate-to-vigorous PA for at least 60 min each day on most days of the week [25]. However, youths with disabilities experience difficulty in meeting this guideline [26] and have a much higher risk of obesity compared with that of their counterparts without IDs [27]. Data from the 1999 to 2010 National Health and Nutrition Examination Survey indicated a lower prevalence of PA and higher prevalence of obesity among US youths (12–17 years of age) with disabilities compared to their counterparts without disabilities [28]. A secondary data analysis of a publicly available dataset from the National Survey of Children’s Health indicated that US youths (6–17 years of age) with special healthcare needs (i.e., functional limitations or service use) were two times less likely to meet PA recommendations (≥60 min of vigorous PA per day) than were youths without special healthcare needs [29]. These figures increased after obesity status was added to the analysis models, indicating a potential additive adverse effect of obesity on participation in vigorous PA in this population. Further analyses of the National Survey of Children’s Health data, that included items characterizing youths (aged 10–17 years) with and without IDs along with information on weight status, yielded similar findings, indicating that the prevalence of obesity is almost twice as high among youths with IDs than it is among youths without IDs [30]. A study reported that youths with IDs (mean age, 13.04 ± 4.45 years; range, 6–23 years) engaged in substantially less PA than did those in other disability groups [31], and recommended that such youths should thus be a target population for PA and health promotion. Collectively, these findings signal the need for further efforts to promote PA among youths with IDs.

Evidence supports the beneficial effect of PA interventions on motor proficiency in younger youths with IDs (aged 6–11 years); however, it is currently unclear whether the effects of PA interventions are effective in improving motor proficiency in older youths with IDs (aged 12–17 years) [32]. To date, only three studies have examined the effects of PA interventions on changes in motor proficiency of older youths with IDs. Işık and Zorba [33] conducted a pretest–posttest control group design on the motor proficiency of 50 youths in the 12–16 age group (mean age, 13.85 ± 5.13 years) with mild and moderate IDs, as determined by three sub-tests of the Bruininks–Oseretsky Test of Motor Proficiency, Second Edition (BOT-2). They [33] reported improved balance, bilateral coordination, and upper-limb coordination in the experimental group following 12 weeks (3 days per week, 60 min per day) of a basic hemsball training program. Gupta et al. [34] conducted a randomized controlled trial to examine the effect of PA training on strength and balance in youths with Down’s syndrome aged 10–14 years. The participants were randomly assigned to experimental (*n* = 12; mean age, 13.50 years) and control groups (*n* = 11; mean age, 13.00 years), and assessed before and after 6 weeks of PA training. Balance was measured using the balance subscale of the Bruininks–Oseretsky Test of Motor Proficiency (BOTMP). The results indicated that the program improved the balance subset, except for in three components (i.e., walking on a line, standing on a balance beam with eyes closed, and stepping over a response stick on the balance beam), of the youths with Down’s syndrome. Kubilay et al. [35] evaluated the effect of an 8-week (5 times per week for 30 min periods) balance training and postural exercise on the function level in 28 youths with mild IDs. The authors [35] observed a significant increase in coordination, functional mobility, and balance of the experimental group (*n* = 14; mean age, 14.28 ± 5.13 years), and concluded that regular balance training and postural exercise may benefit the functional level of youths with IDs. Therefore, additional research is warranted to elucidate whether different PA intervention contents affect various components of motor proficiency in older youths with IDs.

Sufficient studies have indicated that PA interventions can be effective for improving cardiovascular and muscular fitness in adults with IDs [36]. However, few studies have explored PA as a means of improving the physical fitness of youths with IDs. To date, data on the effects of PA intervention addressing physical fitness for youths with IDs are promising. Wang et al. [13] conducted a randomized controlled trial to examine the effect of a 12-week (2 days per week, 60 min per day) school-based PA intervention and to evaluate sustainable effects of the intervention on physical fitness of 48 youths with IDs (aged 12–18 years). This study is ongoing, and the results of improving physical fitness levels in youths with IDs are expected at the end of 2021. Zurita-Ortega et al. [37] conducted a pre-experimental study in a group of 47 individuals with IDs (aged 12–55 years) and reported improved cardiorespiratory endurance following a 12-week (1 day per week, 60 min per day) Kin-Ball program. Kong et al. [38] investigated the effects of 12-week (2 sessions per week, 60 min per session) school-based Tai Chi and aerobic exercise programs in youths with IDs (aged 10–18 years). Compared with the ID youths in the arts/crafts activities as a control condition (*n* = 19), those in the Tai Chi (*n* = 17) and aerobic exercise (*n* = 17) interventions demonstrated similar improvements in cardiorespiratory fitness. In terms of physical fitness outcomes, the aerobic exercise group also exhibited positive effects in abdominal strength and endurance after the 12-week intervention. The authors [38] concluded that two different PA interventions have distinctive advantages on training of physical fitness.

In addition to PA interventions that focus on physical outcome variables (e.g., motor proficiency and physical fitness), Kapsal et al. [39] collected the literature and quantified the effects of PA on the psychosocial health of youths with IDs, and indicated that intervention content and outcome type were significant moderators for both physical and psychosocial variables. This means that in terms of physical health outcomes, the success of a PA intervention for youths with IDs is likely to depend heavily on the specific results (i.e., outcomes) the practitioner is seeking to achieve. By contrast, regarding the effect of PA on psychosocial health, the type of intervention is more important for improving psychosocial health than physical health. Kapsal et al. [39] suggested that practitioners seeking maximal improvements in both the physical and psychosocial health of youths with IDs may wish to focus interventions on participation in sports and the training of sports and movement skills. These interventions could include activities such as basketball skills training, soccer skills training, ball throwing programs, judo training, and table tennis. These activities appear more effective in increasing both physical and psychosocial health than other types of exercise training, such as resistance, aerobic, and balance or core stability training. The enhanced benefits of playing sports could be attributable to the social nature of many of these activities, which contrasts with the solitary nature of resistance exercise and running. This idea accords with the findings of other research on youths with developmental disabilities and suggests that youths with IDs benefit more from physical activities when they are performed in groups [40]. Thus, the review by Kapsal et al. [39] suggests that these types of PA (i.e., participation in sport and training for sports and movement skills) are promising methods for improving the physical and psychosocial health of youths with IDs.

Although not specific to PA participation and adaptive behaviors in secondary school aged youths with IDs, Killeen et al. [41] investigated the extent to which personal factors and adaptive behaviors explain the participation patterns (i.e., play, skill development, active physical recreation, and social activities) of youths with and without a history of preterm birth. Participation patterns were measured using a standardized assessment with satisfied internal consistency and construct validity [42]. The authors [41] reported that all youths tended to participate most frequently in play (e.g., playing with toys, creating a craft) and less frequently in social activities (e.g., playing a board game, going on an outing). Adaptive behaviors measured using the Adaptive Behavior Assessment System, Second Edition (ABAS-II), were observed to have significant positive associations with the intensity of participation in each activity type and the total diversity of participation (i.e., number of activities performed). The strongest effects, as indicated by standardized coefficients, were noted for the aspects of intensity of play, skill development, and total score for diversity of participation. In other words, youths with more adaptive behavior tended to participate more frequently in play- and skill-based activities. However, their study [41] had a cross-sectional design. Thus, the researchers could not conclusively determine whether adaptive behaviors, as measured using the ABAS-II, are the foundation for participation—meaning that better adaptive behaviors enhance youth’s level of participation—or whether youths with higher levels of participation develop better adaptive behaviors. Since both adaptive behaviors and participation levels are more amenable to change than personal factors are, more studies are necessary to disentangle this relationship so that interventions for youths with IDs can be targeted at the appropriate physical (e.g., motor proficiency and physical fitness) and psychosocial (e.g., adaptive functioning) outcome variables.

Floor hockey was first introduced as a Special Olympics sport at the 1970 Special Olympics World Winter Games. Special Olympics is an organization that empowers individuals with IDs to compete physically with their peers and develop friendships with family members and volunteers through athletic competition. People with IDs who participate in this sport activity are called athletes [43]. Special Olympics athletes (ages 8 years old and up) and young athletes (ages 2 to 7) come from more than 170 countries. The goal of Special Olympics is to reach out to people with IDs at any age and in every country to learn new skills, make new friends, and gain in fitness and confidence [43]. Floor hockey is adapted from the games ice hockey and ringette and is typically not a dangerous sport because the game is relatively well-structured. Floor hockey is played on a wooden floor. Teams comprise six players, one of whom is a goalie. The athletes use wooden poles (without blades) as sticks, and the pucks are large felt disks with an open center. Floor hockey events combine both team and individual skills competitions. Meaningful involvement of all teammates ensures a positive and rewarding experience for everyone. Teammates have the ability and opportunity to contribute to the performance of the team. Teammates understand how to combine their skills with those of other athletes, resulting in improved performance by athletes with lower ability levels. The floor hockey training season is usually from November to March each year. More than 500 Special Olympics athletes partake in floor hockey training [44]. The features of floor hockey include an emphasis on skill (e.g., physical and psychosocial) development [41] and group participation [40]. Despite the advantages of the sport, the potential of floor hockey training as a behavioral health intervention in youths with IDs has not been fully elucidated.

Therefore, the present study examined the efficacy of a 12-week (3 days per week, 90 min per day) floor hockey training program in improving the motor proficiency (assessed using the BOT-2) [45], physical fitness (assessed using the Brockport Physical Fitness Test; BPFT) [46], and adaptive development (assessed using the Chinese version of the teacher form of the ABAS-II) [47] of youths with IDs. It was hypothesized that the floor hockey intervention would improve motor proficiency, physical fitness, and adaptive development in this population.

## 2. Materials and Methods

### 2.1. Experimental Design

A total of 54 youths with mild IDs who were aged 15 to 17 years participated in this study, which adopted a nonequivalent pretest–posttest control group design. Each participant was assessed two times: once at study entry to serve as the baseline (T1) and the other after a 12-week intervention to serve as a postintervention assessment (T2). Before the intervention, all participants were screened for eligibility. They were then paired on the basis of age, gender, and comorbidities, and then randomly assigned to an exercise group (EG; male, *n* = 23; female, *n* = 4) or control group (CG; male, *n* = 23; female, *n* = 4). The participants were unaware of which group they had been assigned to, and they were asked to continue partaking in their regular activities during the intervention. Both T1 and T2 assessments were completed within 1 week before and after the intervention.

### 2.2. Participants

The sampling design was purposive, meaning that specific criteria were used to select youths with mild IDs. To be included in this study, participants were required to meet the following inclusion criteria: (a) age of 15 to 17 years and able to follow an exercise intervention independently, and (b) record of an ID diagnosis, with an intelligence quotient score of ≥50 [48]. Exclusion criteria for participants in this study were (a) a diagnosis of Down’s syndrome, (b) attending any structured PA programs for the past 6 months, (c) having contraindications for PA (e.g., severe heart disease), or (d) taking medication that could interfere with the study results. To recruit participants, the principal investigator contacted local ID associations and public schools. Before the study began, a workshop was held by the principal investigator to introduce the purpose of this study, the floor hockey training program designed, the assessments administrated, the procedures involved, and the benefits of participating in the floor hockey intervention. Participants were recruited in a large urban city with a high level of socioeconomic deprivation in southern Taiwan. The participation rate of this study was 100%. All the participants attended inclusive high schools and were placed in comprehensive vocational classes to receive special education services. The research proposal was reviewed and approved by the Institutional Research Ethics Committee for the Protection of Human Subjects of National Changhua University (NCUEREC-103-086). Written informed consent forms were signed by and obtained from all the parents and youths. The descriptive characteristics are listed in Table 1.

### 2.3. Floor Hockey Intervention

During the 12 consecutive weeks of the study, the participants in the EG partook in the floor hockey training program (3 days per week, 90 min per day). Each session was divided into the following categories: (a) warm-up activities (15 min), (b) floor hockey training (45 min), (c) games and physical fitness training (15 min), and (d) cool-down activities (15 min). The current intervention was mainly taken from the Special Olympics Floor Hockey Coaching Guide developed for individuals with IDs [49,50]. The primary coach conducted each intervention session and guided the transitions between the four activities. The activities, content, and goals of the intervention are listed in Table 2.

To maximize quality control, the primary investigator provided a written treatment protocol for conducting the intervention. The primary investigator trained one primary coach and two research assistants before delivering any lessons to ensure that they were aware of the lesson focus and required drills so that each lesson would be delivered according to the specified protocol. The primary coach had been an elite national baseball player for 1 year and had 6 years of experience teaching floor hockey exercise to youths with IDs. The primary coach and research assistants majored in physical education, adapted physical education, or special education. They had teaching experience with individuals with IDs.

Lesson content for the 12 weeks was adapted for the first few sessions; thereafter, a sequence for this training modality was developed and followed. Adapted activities were introduced throughout the intervention according to individual needs (e.g., different learning pace and ability, execution time). Criteria for the adapted activities included being safe for youths with IDs, easy to learn, fun, capable of reaching moderate- to vigorous-intensity PA, and feasible to perform in schools. Some additional practice activities were developed to help participants generalize their skills. In addition, for competitions, emphasis was placed on the rules not being changed to suit the athletes’ special needs. However, the primary coach was permitted to modify the training activities to accommodate each participant’s special needs or adapt sport equipment to assist participants in achieving success. The adaptations and implementation processes were supervised by the primary investigator each session.

### 2.4. Instruments and Procedures

All measurements were conducted in a gym. During the initial assessment, all parents completed a supplemental information form providing demographic data (e.g., age and gender) and a summary of the developmental history of their child.

#### 2.4.1. Motor Proficiency

The motor proficiency of each participant was assessed using the standardized assessment of the BOT-2 [45], which has good validity (i.e., content validity, construct validity, and criterion validity) and reliability (i.e., internal consistency, interrater reliability, and test–retest reliability) to measure fine and gross motor proficiency among youths and young adults with IDs aged 4–21 years [45].

The BOT-2 is divided into four main domains of motor function, each of which comprises two subscales. The four motor-area composites are fine manual control, manual coordination, body coordination, and strength and agility. The eight subscales are fine motor precision, fine motor integration, manual dexterity, upper-limb coordination, bilateral coordination, balance, running speed/agility, and strength. The four subdomain scores were added together to generate a total motor composite score, which represents overall motor proficiency (53 items, 8 subtests, and 4 motor-area composites; score range = 0–320 points). Approximately 10 to 15 min were needed to administer the activities in each composite, with the complete form taking approximately 40 to 60 min to administer. The BOT-2 subtest scores are age-adjusted scaled scores with a mean of 15 and a standard deviation of 5, whereas the BOT-2 composite scores are standard scores (derived from summing the subtest scale scores and converting them into a quotient) with a mean of 100 and a standard deviation of 15. Scaled scores were converted into standard scores.

#### 2.4.2. Physical Fitness

Body mass and standing height were assessed as anthropometric parameters. Body mass was measured using a bioelectrical impedance analyzer (MFBIA8, InBody 720, Biospace) while the participant was wearing light clothing and recorded to the nearest 0.1 kg. Standing height in bare feet was evaluated and recorded to the nearest 0.1 cm. Body mass index (BMI, in kg/m^2^) was computed as the participant’s weight divided by the square of their height.

The BPFT [46] was used to assess the physical fitness components of the participants and included: (a) the 20 m Progressive Aerobic Cardiovascular Endurance Run (PACER) test, (b) a modified curl-up test, (c) an isometric push-up test, and (d) the back-saver sit-and-reach test. The raw scores were used for data analysis. The BPFT is a health-related, criterion-referenced physical fitness test for youths aged 10 to 17 years, and the BPFT manual includes separate instructions for youths with and without disabilities. The validity of the BPFT was demonstrated through confirmation of the concurrent, construct, and content validity of each item for each disability involved [46].

The 20 m PACER multistage shuttle run was used to estimate an individual’s VO_2_ max and thus measure their cardiovascular fitness. In the PACER test, the participants were instructed to run as long as possible back and forth across a 20 m distance at a specified pace, which became progressively faster each minute. One trial was conducted, and the test was terminated when the participant withdraws from the test, or when the participant could no longer maintain the pace for two consecutive laps. The participant’s score was recorded as the number of completed laps. The PACER test has demonstrated acceptable concurrent validity and criterion-referenced validity for measuring and estimating VO_2_ max [51,52].

Abdominal muscular strength and endurance was measured using the modified curl-up test. At the beginning of the test, the participants lay in a supine position while maintaining approximately 140° knee flexion with their feet placed flat on the floor, their legs held slightly apart, and their hands placed on the front of the thighs. During the test, the participants contract their abdominal musculature until their hands slide along their thighs and their fingertips slide at least 10 cm to the patellae, and then return to their starting position. Correct curl-ups also include that both of their hands slide up at the same time to the right and left knee caps, and their fingers touch closely to their legs. One test trial was conducted, and the participants completed as many curl-ups as possible at a rate of one curl-up every 3 s. The tester verbally counted the number of curl-ups completed, and the test was terminated at 75 curl-ups or when the participant was no longer able to perform curl-ups by using the appropriate form.

Upper-body muscular strength and endurance was measured using the isometric push-up test, which was scored as the participants’ attempt to hold a raised push-up position for as long as 40 s. The participants assumed a front-leaning rest position with their hands positioned directly below their shoulders, arms fully extended, the whole body in a straight line, and the toes touching the floor or mat. The test was terminated at 40 s or when the correct front-leaning rest position was no longer held. One test trial was conducted, and the time was recorded to the nearest 0.1 s.

The hamstring and lower-back muscles of flexibility were assessed using the back-saver sit-and-reach test. Participants sat down at the sit-and-reach box with one leg straight against the end of the test box, and the other leg bent placed next to the knee of the straight leg. In the test, participants were instructed to push the sliding metal with both hands toward the box, performed four times, holding the final reach position for at least 1 s. After measurements had been obtained for one side, the participants switched leg positions, and then the assessments were repeated. Participants performed this test once for each leg, and the distance was recorded in centimeters.

#### 2.4.3. Adaptive Development

The traditional Chinese version of the teacher form of the ABAS-II [47], originally developed by Harrison and Oakland [53], was used to measure the participants’ overall adaptive development. The teacher form is used for individuals aged 6–17 years and contains 172 items. Items are rated on the following scale: 0 (is unable), 1 (never when needed), 2 (sometimes when needed), and 3 (always when needed). The traditional Chinese version of the ABAS-II measures nine skill areas and yields four composite scores for the following skill categories: conceptual (e.g., telling teachers, friends, or others your favorite activities; reading important documents, for example, registration notice or leave policy), social (e.g., inviting and letting others play games or other interested activities, saying please when asking for a favor), practical (e.g., choosing appropriate clothing based on weather, asking for help when someone is hurt), and general adaptive skills. The general adaptive composite score is an overall standard score that summarizes an individual’s adaptive functioning across all skill areas.

The teacher form of the Chinese version of the ABAS-II has satisfied test–retest reliability for three of the composite scores (i.e., conceptual, practical, and general adaptive), with coefficient values ranging from 0.62 to 0.74, but yielded a coefficient value of only 0.40 for the social composite score. The test–retest reliability was high, ranging from 0.84 to 0.91, and the internal consistency was excellent, ranging from 0.97 to 0.99. Studies have provided support for the construct and discriminant validity of the Chinese version of the ABAS-II, as detailed in the user manual [47].

### 2.5. Data Analysis

Preliminary data analyses were conducted to determine the possibility of gender effects on all outcome variables. No effects were identified; therefore, gender was not included in subsequent analyses. To assess the effects of the floor hockey intervention, analysis of variance (ANOVA) with a 2 (time: T1 versus T2) × 2 (group: EG versus CG) mixed-model factorial design was conducted for the outcome variables. The calculated effect sizes are reported as partial eta squared (*η*^2^). If an interaction effect was significant, the least significant difference post hoc test was performed to examine between-group differences at each time point, as well as within-group differences (T2 vs. T1). A follow-up analysis was conducted, which involved testing for simple main effects with significant interaction effects (i.e., group differences at each time point and within-group differences). All statistical analyses were performed using SPSS (version 20.0; IBM Corp., Armonk, NY, USA). To control for possible type I error inflation caused by multiple comparisons in repeated-measures ANOVA tests, the alpha level was set at *p* < 0.01.

## 3. Results

### 3.1. Effect of the Floor Hockey Intervention on Motor Proficiency

The results for motor proficiency outcomes are presented in Table 3. No differences were noted between the EG and CG at the time of study enrolment (T1) in terms of any of the demographic (Table 1) or motor proficiency outcome variables (Table 3).

The ANOVA results indicated that the group assignment had no main effect on any of the BOT-2 composite scores (Table 4). Time was discovered to have a significant main effect on two motor-area composite scores (i.e., manual coordination: *F* = 18.70, *p* < 0.01, *η*^2^ = 0.26; body coordination: *F* = 40.61, *p* < 0.01, *η*^2^ = 0.44) and the total motor composite score (*F* = 53.00, *p* < 0.01, *η*^2^ = 0.51). Specifically, the scores were significantly higher at T2 (manual coordination, 38.82 ± 1.16; body coordination, 44.35 ± 1.06; total motor composite score, 37.96 ± 0.74) than at T1 (manual coordination, 35.89 ± 1.01; body coordination, 37.63 ± 1.15; total motor composite score, 35.13 ± 0.73). Furthermore, significant group × time interaction effects were observed for manual coordination (*F* = 9.06, *p* < 0.01, *η*^2^ = 0.15), body coordination (*F* = 16.45, *p* < 0.01, *η*^2^ = 0.24), and the total motor composite score (*F* = 7.36, *p* < 0.01, *η*^2^ = 0.12).

**Figure 1:**
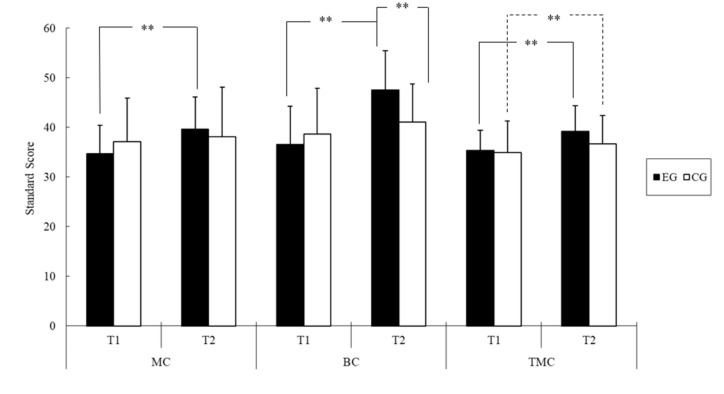
The BOT-2 standard score of two groups of youths with IDs before (T1) and after the intervention (T2); ** *p* < 0.01 (Abbreviations: MC = manual coordination; BC = body coordination; TMC = total motor composite; EG = exercise group; CG = control group).

As shown in Figure 1, a follow-up analysis of the simple main effects revealed that individuals in the EG exhibited higher body coordination composite scores after the floor hockey intervention (T2) compared with individuals in the CG (+6.48, *F* = 9.37, *p* < 0.01). Additionally, participants in the EG had a significantly higher score at T2 than at T1 (manual coordination: +4.96, *F* = 24.81, *p* < 0.01, *η*^2^ = 0.49; body coordination: +11.00, *F* = 42.99, *p* < 0.01, *η*^2^ = 0.62; total motor composite score: +3.78, *F* = 49.46, *p* < 0.01, *η*^2^ = 0.66). Participants in the CG were discovered to have a significantly improved total motor composite score (+1.78, *F* = 10.53, *p* < 0.01, *η*^2^ = 0.29) at T2 compared with T1.

### 3.2. Effect of the Floor Hockey Intervention on Physical Fitness

The results for physical fitness performance are listed in Table 3. The ANOVA results revealed a significant main effect of time on scores in the PACER test (*F* = 14.91, *p* < 0.01, *η*^2^ = 0.22) and modified curl-up test (*F* = 13.48, *p* < 0.01, *η*^2^ = 0.21), as shown in Table 4. These scores were significantly higher at T2 (PACER, 25.74 ± 1.56; curl-up, 36.61 ± 2.93) than at T1 (PACER, 21.13 ± 1.59; curl-up, 28.35 ± 2.58). No significant between-group differences were observed for any of the physical fitness scores. Furthermore, scores on the PACER test (*F* = 8.41, *p* < 0.01, *η*^2^ = 0.14) and modified curl-up test (*F* = 10.96, *p* < 0.01, *η*^2^ = 0.17) were observed to have a significant interaction effect (Table 4).

**Figure 2:**
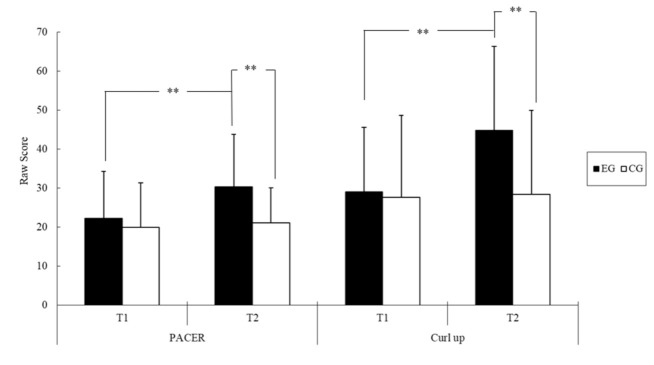
The physical fitness score of two groups of youths with IDs before (T1) and after the intervention (T2); ** *p* < 0.01 (Abbreviations: PACER = Progressive Aerobic Cardiovascular Endurance Run; EG = exercise group; CG = control group).

Follow-up analysis of the simple main effects revealed that scores in both the PACER (*F* = 8.70, *p* < 0.01) and modified curl-up (*F* = 7.75, *p* < 0.01) tests were higher in the EG than in the CG at T2 (Figure 2). Regarding within-group differences between T1 and T2, scores in the PACER (+8.07, *F* = 20.14, *p* < 0.01, *η*^2^ = 0.44) and modified curl-up (+17.70, *F* = 25.92, *p* < 0.01, *η*^2^ = 0.50) tests were significantly higher at T2 than at T1 in the EG, but were similar between the two time points in the CG.

### 3.3. Effect of the Floor Hockey Intervention on Adaptive Development

The results for adaptive development are listed in Table 3. The ANOVA results revealed that group assignment did not have any significant main effects on scores for any of the adaptive development indices, whereas time exerted significant main effects on scores for all of these indices (Table 4). Specifically, the scores were significantly higher at T2 (conceptual composite, 93.67 ± 1.82; social composite, 96.17 ± 2.00; practical composite, 99.48 ± 1.84; general adaptive composite, 96.44 ± 1.85) than at T1 (conceptual composite, 86.16 ± 1.80; social composite, 87.30 ± 1.84; practical composite, 93.04 ± 2.08; general adaptive composite, 88.74 ± 1.80). Significant group × time interactions were noted for the conceptual composite score (*F* = 9.44, *p* < 0.01, *η*^2^ = 0.15) and social composite score (*F* = 12.75, *p* < 0.01, *η*^2^ = 0.20).

**Figure 3:**
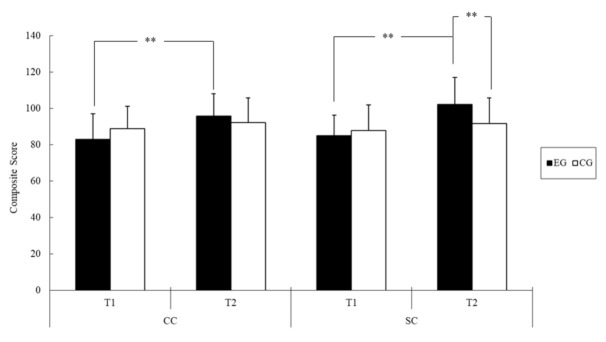
The adaptive development composite score of two groups of youths with IDs before (T1) and after the intervention (T2); ** *p* < 0.01 (Abbreviations: CC = conceptual composite; SC = social composite; EG = exercise group; CG = control group).

A follow-up analysis of the simple main effects revealed that the social composite score after the intervention was higher in the EG (+10.66, *F* = 7.42, *p* < 0.01) than in the CG (Figure 3). Regarding within-group differences between T1 and T2, the EG had a significantly higher conceptual composite score (+12.82, *F* = 44.75, *p* < 0.01, *η*^2^ = 0.63) and social composite score (+17.37, *F* = 36.37, *p* < 0.01, *η*^2^ = 0.58), whereas these scores were similar between the two time points in the CG.

## 4. Discussion

To our knowledge, this is the first time that floor hockey training has been employed to target changes in the motor proficiency, physical fitness, and adaptive development of youths with mild IDs. The main results of this study indicate that in youths with IDs, this intervention results in increased scores for most indicators of motor proficiency (i.e., manual coordination, body coordination, and total motor composite scores), physical fitness (i.e., cardiovascular fitness, abdominal strength, and endurance scores), and adaptive development (i.e., conceptual and social composite), but does not cause significant changes in fine motor control, strength and agility, upper-body muscular strength and endurance, flexibility, or practical and general adaptive composite scores.

Compared with youths with typical development between the ages of 7 and 12 years, youths with IDs tend to have significantly lower motor proficiency skill levels in terms of fundamental movement [6] and locomotor and object control [5,6,8]. A systematic review summarized the findings of 14 studies concerning youths with IDs aged 5 to 22 and revealed that PA interventions are efficient in improving fundamental movement skills (i.e., locomotion, object control, and balance skills) [32]. Most of the studies focused on the balance skills of youths with mild IDs and offered strong support for the effect of PA interventions on balance skills. These positive effects of PA interventions are consistent with the findings of empirical research concerning the effects of PA interventions on motor proficiency in youths without disabilities [54,55]. The interventions were effective for youths with IDs when the intervention duration was 6–24 weeks and when the training sessions were held three times per week for a duration of 20–60 min for these youths. Such results were obtained when researchers employed adapted play training [56], virtual reality therapy [57,58], Hemsball game skills [33], strength and balance training [34], or Swiss ball exercises [35]. Additionally, several studies on youths with IDs [37,59,60] have reported improvements in motor proficiency following long-term PA interventions involving Drums Alive Kids Beats programs [59], Kin-Ball exercises [37], and PA promotion with a parental involvement component [60]. Results of the current study indicate that the floor hockey training program improves manual coordination through floor hockey activities (e.g., skills and drills), enhances body coordination through strength and cardiovascular exercises (e.g., skills training, drills, and games), and therefore promotes the development of overall motor proficiency. The cumulative effects of a series of training steps that are goal-directed, structured, progressive, and interrelated may account for the significant improvements in motor proficiency observed in this study [61,62]. For example, once a participant had learned to control the stick, they were presented with dynamic tasks that were more challenging, such as moving the puck without looking down at it, passing the puck to other players, moving around the court while controlling the puck, looking in one direction while passing the puck in another direction, and scoring goals. After mastering these tasks, the participants were better able to utilize sensory perceptions to understand the outcome of movements (feedback), anticipate upcoming events (feedforward), and plan alternative strategies [61,62].

Regarding physical fitness, some intervention studies of youths with IDs have reported global changes in health-related physical fitness components [13,37,38,60,63]. Youths with IDs in the present study were discovered to have significant improvements in physical fitness components, namely cardiorespiratory fitness and abdominal strength and endurance, after the 12-week floor hockey intervention (Table 4 and Figure 2). Findings accord with several intervention studies on youths with IDs and typically developing youths that have revealed positive effects of PA interventions on several physical fitness outcomes in this population [13,37,38,60,63,64]. These improvements in the physical fitness of the participants with IDs may be attributable to improvements in motor proficiency due to increases in PA (i.e., floor hockey training). Two studies conducted in Asia on samples of more than 400 youths with IDs identified significant correlations of motor proficiency with muscular strength and endurance and cardiorespiratory fitness [65,66]. The positive findings of the current study echo those of early studies [13,37,38,60,63], synthesizing evidence regarding the effects of PA training on physical fitness levels in youths with IDs. Our results also extend the findings of these early studies by indicating that floor hockey practice can improve not only physical fitness but also other motor proficiency and adaptive functioning outcomes among youths with IDs. Nevertheless, training was not observed to have any effects on upper-body muscular strength or endurance, as assessed using the isometric push-up test. This nonsignificant finding for parameters related to physical fitness may be attributable to a ceiling effect, because 85% (*n* = 23) of the participants in each group could sustain a raised push-up position for 40 s, the maximum considered. Further investigation is necessary to determine whether floor hockey training is effective for improving upper-body muscular strength and endurance in youths with IDs. Future studies can also include other measures, such as the handgrip strength test with a hand dynamometer that has an adjustable grip.

Regarding adaptive development, an insufficient number of published studies on the effectiveness of PA interventions have investigated the adaptive functioning of youths with IDs. Levels of adaptive functioning, which is an individual’s ability to perform age-appropriate skills in domains important in daily life, are low among individuals with IDs [2,67]. Research has indicated that a positive correlation exists between motor proficiency (i.e., visual motor integration) and adaptive functioning in youths with disabilities [68]. This information can guide PA interventions targeting motor proficiency for clinical practice to improve adaptive development in youths with IDs. The present study further evaluated the association between motor proficiency and adaptive development, and the results confirm that the two are positively related. For many people with diverse abilities, the disabilities they experience often require special approaches to education or other accommodations [69]. The current comprehensive independent study was conducted to evaluate the positive effects of a floor hockey training program, which has multi-modular disciplines (skill-based training, game involvement, strength training and conditioning, and peer modeling), on motor proficiency and physical fitness, as well as adaptive behavior and learning among individuals with diverse abilities. Evidence strongly suggests that the floor hockey training program employed in the present study has a high level of applicability, not only cross-culturally but also across socioeconomic barriers; moreover, this program is unique in that anyone can participate, regardless of their age or ability. As mentioned, the results and findings provide research-based evidence that the floor hockey training program—which integrates components of strength and endurance training, play and adaptive skill development, and highly coordinative physical movements with emotional elements in an enriched environment—resulted in significant and therapeutically proven improvements for the majority of the participants in this study.

Although the results of the present study are promising, they should be regarded as preliminary findings and interpreted with caution in light of the limitations of this study. First, the small sample size, small age range, and unevaluated differences in family socioeconomic status and participant’s PA level might have influenced findings and limit generalization. Second, the sample was purposive, and the results of this study are not generalizable to youths with severe or profound IDs because different outcomes may be obtained for individuals with different ID levels. Third, the effect sizes of the intervention effect were small (<0.24), and this should be noted. Fourth, the results of this study may not be applicable to youths studying in special schools because school settings may differ between mainstream schools and special schools. Fifth, determining which characteristics and contextual features of the floor hockey intervention achieve most of the improvements in motor proficiency, physical fitness, and adaptive development in youths with IDs is impossible. Sixth, the long-term sustainability of the effects of the floor hockey intervention remains unknown. Future studies should include larger samples and consider various ID severities, school settings, PA levels, and family socioeconomic status of youths with IDs. Moreover, a follow-up study could conduct a comparison of training programs with activities that directly aim at adaptive development with the current intervention program, which could separate the factors contributing to the physical and psychosocial effects. Future research is warranted to establish the applicability of the current findings to individuals at different stages of the lifecycle through replication studies. Finally, investigating the possible sustained intervention effects in improving outcome variables may yield useful findings.

## 5. Conclusions

In conclusion, the current study provided preliminary evidence that the developed 12-week floor hockey training program has positive effects on the motor proficiency, physical fitness, and adaptive development of youths with mild IDs. These benefits further elucidate the potential applications of floor hockey training as a complementary intervention for motor skill rehabilitation and treatment for adaptive behavior disturbances, and for increasing the physical fitness levels of youths with IDs. Additional research should be conducted to replicate and extend these findings. Such investigations could reveal the PA components that influence motor, physical, and behavioral function outcomes and determine the efficacy of PA interventions involving floor hockey exercise for individuals with IDs.

## Figures and Tables

**Table 1:** Descriptive characteristics of the participants.

	Exercise Group *n* = 27	Control Group *n* = 27	*t*	*p*
Age (years)	16.59 ± 0.56	16.65 ± 0.63	−0.37	0.71
Height (cm)	167.80 ± 8.13	165.98 ± 9.58	0.75	0.46
Weight (kg)	66.43 ± 18.53	67.43 ± 17.01	−0.21	0.84
BMI (kg/m^2^)	23.36 ± 5.42	24.42 ± 5.68	−0.70	0.49
Gender (*n*, %)				
Male	23 (85.19%)	23 (85.19%)	--	--
Female	4 (14.81%)	4 (14.81%)	--	--

Note. Means ± standard deviations (M ± SD). BMI = body mass index.

**Table 2:** Activities of a 90 min floor hockey lesson throughout the 12-week intervention.

Activities	Length	Weeks	Key Tasks to Be Delivered	Goal
Warm-up activities	15 min	1–12	Slow aerobic walk/jog/fast walk/run/Mach sprint drills/stretching	⮚ Raises body temperature ⮚ Increases metabolic/heart/respiratory rate ⮚ Prepares the muscles and nervous system for exercise
Floor hockey activities	45 min	1–2	⮚ Stick handling and drills/passing and drills	⮚ Motor proficiency development ⮚ Physical fitness improvement ⮚ Adaptive skill development ⮚ Friendship development
		3–4	⮚ Receiving and drills/stick checking and drills	
		5–6	⮚ Face-off and drills/shooting on goal and drills	
		7–8	⮚ Defense and drills/offense and drills	
		9–10	⮚ Goal tending and drills	
		11–12	⮚ Cross training/competition rules	
Floor hockey skill-based games	15 min	1–12	⮚ Hockey dodge ball/individual and team races ⮚ Skills competition (i.e., longest slide/fastest skater/fastest skills course time/most accurate shooter/most accurate passer/hit the dot/angry birds/3 on 3 or 2 on 2 tournament) ⮚ Progressive aerobic/muscular training	⮚ Physical fitness improvement ⮚ Adaptive skill development ⮚ Friendship development
Cool-down activities	15 min	1–12	Slow aerobic jog/run/light stretching/flexibility training	⮚ Gradually lowers heart rate and body temperature ⮚ Removes waste from muscles ⮚ Social interaction and communication

**Table 3:** Changes in motor proficiency, physical fitness, and adaptive development of the participants following 12 weeks of intervention.

	T1		Exercise Group		Control Group	
	*t*	*p*	T1	T2	T1	T2
**BOT-2**						
Fine motor control	0.90	0.38	35.70 ± 4.87	35.04 ± 6.54	34.48 ± 5.15	35.93 ± 5.20
Manual coordination	−1.25	0.22	34.63 ± 5.81	39.59 ± 6.56	37.15 ± 8.70	38.04 ± 10.07
Body coordination	−0.90	0.37	36.59 ± 7.70	47.59 ± 7.88	38.67 ± 9.20	41.11 ± 7.69
Strength and agility	1.68	0.10	43.15 ± 6.77	44.04 ± 6.45	40.07 ± 6.70	40.96 ± 6.98
Total motor composite	0.28	0.78	35.33 ± 4.10	39.22 ± 5.15	34.93 ± 6.38	36.70 ± 5.72
**Physical fitness**						
PACER (*n*)	0.71	0.48	22.26 ± 12.02	30.33 ± 13.49	20.00 ± 11.31	21.15 ± 8.93
Modified curl-up (*n*)	0.28	0.78	29.07 ± 16.49	44.78 ± 21.60	27.63 ± 21.08	28.44 ± 21.52
Isometric push-up (s)	0.27	0.79	37.81 ± 7.87	38.63 ± 5.09	37.25 ± 7.45	36.67 ± 9.71
Right leg sit-and-reach (cm)	0.72	0.47	28.85 ± 6.59	28.09 ± 8.90	27.14 ± 10.36	28.72 ± 10.39
Left leg sit-and-reach (cm)	−0.46	0.65	27.07 ± 7.72	27.64 ± 9.29	28.20 ± 10.18	28.69 ± 10.04
**Adaptive development**						
Conceptual composite score	−1.66	0.10	82.89 ± 14.20	95.70 ± 12.42	88.89 ± 12.31	92.22 ± 13.55
Social composite score	−0.86	0.39	84.96 ± 11.28	102.33 ± 14.66	87.96 ± 14.12	91.67 ± 14.11
Practical composite score	0.17	0.81	92.96 ± 16.45	103.22 ± 11.28	92.26 ± 13.73	96.59 ± 15.08
GAC score	−0.32	0.75	87.67 ± 14.06	100.04 ± 12.30	88.81 ± 12.19	93.85 ± 14.19

Note. BOT-2 = Bruininks–Oseretsky Test of Motor Proficiency, Second Edition; PACER = Progressive Aerobic Cardiovascular Endurance Run; GAC = general adaptive composite.

**Table 4:** Results from two-way ANOVA with repeated measures on one factor (time).

	Time Effect			Group Effect			Interaction Effect		
	*F*	*p*	*η* ^2^	*F*	*p*	*η* ^2^	*F*	*p*	*η* ^2^
**BOT-2**									
Fine motor control	0.35	0.56	0.01	0.02	0.90	0.00	2.59	0.11	0.05
Manual coordination	18.70	0.00	0.26	0.06	0.82	0.00	9.06	0.00	0.15
Body coordination	40.61	0.00	0.44	1.28	0.26	0.02	16.45	0.00	0.24
Strength and agility	2.78	0.10	0.05	3.08	0.09	0.06	0.00	1.00	0.05
Total motor composite	53.00	0.00	0.51	0.52	0.47	0.01	7.36	0.00	0.12
**Physical fitness**									
PACER (*n*)	14.91	0.00	0.22	3.87	0.06	0.07	8.41	0.00	0.14
Modified curl-up (*n*)	13.48	0.00	0.21	3.11	0.08	0.06	10.96	0.00	0.17
Isometric push-up (s)	0.01	0.93	0.00	0.58	0.45	0.01	0.30	0.59	0.01
Right leg sit-and-reach (cm)	0.20	0.66	0.00	0.05	0.82	0.00	1.60	0.21	0.03
Left leg sit-and-reach (cm)	0.29	0.60	0.01	0.21	0.65	0.00	0.00	0.96	0.00
**Adaptive development**									
Conceptual composite score	27.39	0.00	0.35	0.15	0.70	0.00	9.44	0.00	0.15
Social composite score	30.32	0.00	0.37	1.46	0.23	0.03	12.75	0.00	0.20
Practical composite score	14.85	0.00	0.22	1.17	0.28	0.02	2.45	0.12	0.05
GAC score	26.50	0.00	0.34	0.63	0.43	0.01	4.70	0.04	0.08

Note. BOT-2 = Bruininks–Oseretsky Test of Motor Proficiency, Second Edition; PACER = Progressive Aerobic Cardiovascular Endurance Run; GAC = general adaptive composite.

## Data Availability

Not applicable.
